# Peripheral T-Cell Lymphoma: Review and Updates of Current Management Strategies

**DOI:** 10.1155/2010/624040

**Published:** 2010-12-30

**Authors:** Tiffany Tang, Kevin Tay, Richard Quek, Miriam Tao, Soo Yong Tan, Leonard Tan, Soon Thye Lim

**Affiliations:** ^1^Department of Medical Oncology, The National Cancer Centre Singapore, 11 Hospital Drive, Singapore 169610; ^2^Department of Pathology, Singapore General Hospital, Outram Road, Singapore 169608

## Abstract

The classification of T-cell and natural-killer- (NK-) cell lymphomas has been updated in the 4th edition of the World Health Organization (WHO) classification of tumors of the haematopoietic and lymphoid tissue published in 2008. Based on recent epidemiological studies, NK-cell lymphomas occur almost exclusively in Asia and South America, although T-cell lymphomas appear to occur in the East as commonly as in the West. Due to the low prevalence of this disease, diagnosis and optimal treatment of patients have not been studied prospectively in large randomized trials. Nevertheless, there has been development in the understanding of T-cell lymphomas and how they should be managed; FDG-PET emerges as an increasingly important tool in diagnosis, gene-expression signatures may aid with prognostication in the future, and novel therapies are currently being studied to improve outcomes in T-cell lymphomas. More work, however, needs to be done, and international collaboration will be pertinent to deriving meaningful results from future clinical studies.

## 1. Introduction

T-cell lymphoma and natural-killer- (NK-) cell lymphoma represent the smaller subsets of non-Hodgkin's lymphoma (NHL) that appear to have a geographical predilection to Asia. In Europe and North America, T-cell and NK-cell lymphoma account for 5–10% of all cases of NHL whilst in Asia, this percentage is as high as 24% [1]. T-cell lymphomas, as a group, carry a poorer prognosis compared to their B cell counterpart [2]. In the subgroup of patients with a low international prognostic index (IPI) score of 1-2, 5-year overall survival (OS) was 55% in those with T-cell lymphomas and 71% in those with B-cell lymphomas, and this difference in survival was also reflected in patients with higher IPI scores [3]. T-cell lymphomas, however, represent a heterogeneous group of diseases with variations in clinical characteristics, prognosis and response to treatment. This paper reviews the developments in the understanding of mature peripheral T-cell lymphoma (PTCL) and its management. 

### 1.1. WHO Classification of T-Cell Lymphoma

The 4th edition of the World Health Organization (WHO) classification of tumours of the haematopoietic and lymphoid tissue published in 2008 builds upon the previous edition published in 2001, and reclassifies clinicopathological entities based on better understanding from research in the last few years [4]. The WHO classification now contains 22 different T-cell lymphomas, 7 more than the previous classification. These can be subclassified according to whether they are predominantly nodal, extranodal, cutaneous, or leukemic (Table 1). Two new rare EBV-related T-cell diseases affecting children in Asia or Central/South America are recognized in this classification. These are systemic EBV T-cell lymphoproliferative diseases of childhood and hydroa vacciniforme-like lymphoma. Furthermore, anaplastic large-cell lymphoma (ALCL) ALK−positive (ALK+) is now recognized as a distinct entity from the ALCL ALK-negative (ALK−) lymphomas, which occurs in an older population, and which bears a far worse prognosis [5]. To eliminate redundancy, precursor T-lymphoblastic lymphoma/leukaemia has lost its “precursor” prefix as the term “lymphoblastic” implies this. Finally, three new variants of cutaneous T-cell lymphoma (CTCL) were introduced, and these are primary cutaneous aggressive epidermotropic CD8+ cytotoxic T-cell lymphoma, primary cutaneous gamma-delta T cell lymphoma and primary cutaneous small/medium CD4+ T-cell lymphoma.

### 1.2. Epidemiology

PTCL was thought to occur more frequently in Asia. Rüdiger et al. reported frequencies of PTCL in Vancouver to be 1.6% of all NHL compared to 18.3% in Hong Kong [6]. The international PTCL and NKL project reported PTCL and NKL rates of 5–10% in Western countries compared to 10–20% in Asian countries [5]. 

It the study, it was also interesting that the incidence of ALCL (both ALK+ and ALK−) in North America (24%) was almost four-times higher than in Asia (6%). Data from Au et al. and our own institution's suggest that the rate of PTCL in the East may actually be similar to the West [7]. The perceived higher rates of PTCL in the East could perhaps be due to the higher incidence of NKL in the East and the differences in diagnostic evaluation [7]. In their institution, they evaluated 148 consecutive cases of TCL and NKL and found that these accounted for 10.1% and 6.5% of all NHL, respectively. We evaluated a total of 780 patients with malignant lymphomas from 2002 to 2006 and found that extranodal NKL and PTCL comprised 5.0% (39/780) and 7.4% (58/780) of all cases [8].

### 1.3. Clinical Characteristics of TCL

Peripheral T-cell lymphoma, not otherwise specified (PTCL-NOS), Angioimmunoblastic T-cell lymphoma (AITL), and ALCL are the most common subtypes and account for up to 74% of all T-cell lymphomas [5]. AITL tends to occur in elderly patients, and patients often present with disseminated lymphadenopathy, hepatosplenomegaly, and autoimmune phenomena [9]. ALCL ALK+ typically occurs in young men and presentation can be nodal or extranodal, involving the skin, bone, soft tissues, lung, and liver [10]. On the other hand, ALK*‒* ALCL tends to occur in an older population of patients, the presentation is usually nodal, and the disease runs a more aggressive clinical course [11]. PTCL-NOS, represents a heterogeneous category of nodal and extranodal T cell lymphomas that cannot be grouped into defined entities. Most present with peripheral lymph-node enlargement, B symptoms and at diagnosis, the disease is advanced [12]. 

Other uncommon T-cell lymphomas include enteropathy-associated T-cell lymphoma (EATL) [13], adult T-cell leukaemia/lymphoma (ATLL) [14], hepatosplenic T-cell lymphoma (HSTL) [15], and subcutaneous panniculitis-like T-cell lymphoma (SPTCL) [16]. It is now recognized that EATL lymphoma consists of two distinct forms: classical or type I EATL and type II EATL. Type I EATL is associated with coeliac disease while type II EATL occurs in Asia and is not associated with coeliac disease [13]. ATLL is caused by infection with the human T-cell-lymphotropic virus type 1 (HTLV-1), and is rare outside HTLV-I-endemic areas such as the Caribbean, Japan, and parts of central Africa [14]. HSTL is rare; patients present with hepatosplenomegaly and systemic symptoms, and in 20% it occurs in the context of chronic immune suppression [15]. SPTCL occurs more commonly in women, and is associated with autoimmune conditions such as systemic lupus erythematosus [16].

### 1.4. Diagnostic Workup of PTCL

The histopathological diagnosis of PTCL can prove to be a challenge even for the experienced pathologist. In addition to standard histological analysis and immunophenotyping, clinical information is important in accurately classifying the TCL. To highlight this point, in the same international T-cell lymphoma study, a change in diagnosis of PTCL-NOS to ATLL (Adult T-cell lymphoma/leukaemia) occurred in 38.7% of cases after pathologists were informed of the patient's HTLV-1 (human T-cell leukaemia virus) status [5].

The standard panel of immunostains used in the workup of a T-cell lymphoma includes CD20, CD2, CD3, CD4, CD5, CD8, CD30, CD56, TCR-*β*, TIA-1 (T-cell intracellular antigen 1), Ki67, and in situ hybridization assay for EBV (Epstein-Barr virus) encoded RNA (ribonucleic acid). PTCL-NOS describes a heterogeneous population of lymphomas of the T-cell lineage, but which does not fulfill criteria to be classified into the other categories of TCL. Most cases are positive for CD4, TCR*αβ* is expressed and CD5 and 7 are frequently downregulated [12]. AITL is characterized by a polymorphous infiltration of the lymph node with prominent proliferation of high-endothelial venules and follicular dendritic cells. CD3, CD4, CD10, BCL6, PD1, and CXCL13 are typically positive, and EBV-positive B cells are nearly always present [9]. AITL is closely related to follicular helper T cells and the follicular variant of PTCL, although they differ genetically [17]. ALCL, ALK+ and ALCL, ALK− appear similar histologically. There are morphological variations within ALCL but all have hallmark cells, which are cells with eccentric, horseshoe, or kidney-shaped nuclei, often with eosinophilic region near the nucleus. In both subtypes, CD30, CD2, and CD4 and cytotoxic molecules including that of perforin, granzyme B, and TIA-1 are positive. In ALCL, ALK+, the epithelial membrane antigen (EMA) is characteristically present, and CD3 is negative in 75% of cases. In ALCL, ALK−, EMA is positive only in a small proportion of patients, and CD3 is positive [10, 11] (Table 2).

As mentioned above, Type II EATL is distinct to classical EATL. In classical or type I EATL, the tumour ulcerates through the wall of the mucosa, and the intestinal mucosa surrounding the tumour shows enteropathy comprising villous atrophy, crypt hyperplasia, and increased lamina propria lymphocytes and plasma cells. There are large cytotoxic T cells that often express CD103 and lose both CD4 and CD8 markers, and the intraepithelial lymphocytes adjacent may have an identical immunophenotype. In type II EATL, neoplastic cells are more monotonous with a distinctive CD3+, CD8+, and CD56+ phenotype. There are increased intraepithelial lymphocytes, but not the villous atrophy or crypt hyperplasia typically seen in celiac disease or classical EATL [13].

### 1.5. Initial Assessment and Staging of T-Cell Lymphomas

Staging of PTCL is similar to that of other NHL and involves a complete physical examination and routine laboratory tests such as a complete blood count, renal-function test, liver-function test, and serum lactate dehydrogenase level, unilateral or bilateral bone marrow examination (aspirate, trephine, flow cytometry), and radiological imaging with either computerised tomography (CT) scanning or FDG PET/CT (fluorodeoxyglucose positron emission tomography) assessment. 

## 2. FDG PET/CT

PET scans have an established role in the staging and assessment of disease response in B-cell lymphomas, with the ability to differentiate between fibrosis and active disease in a residual mass as well as in prognostication [18]. In contrast, the role of PET in T-cell lymphomas continues to evolve. A recent review of 135 patients with T-cell lymphomas who underwent PET examination at diagnosis or response assessment reported variations in PET-positivity and maximum standardized uptake value (SUV) across the different T-cell-lymphoma subtypes. PET positivity ranged from 50% in cutaneous ALCL to 78% in AITL to 100% in ATLL. Of note, 29% of patients had diseases that were not picked up on diagnostic CT of the neck, chest, abdomen, and pelvis. The sites that were missed included cutaneous, subcutaneous, muscular masses of the scalp, upper and lower extremities, and lymphadenopathy in the epitrochlear and popliteal regions, indicating perhaps a role for PET imaging from vertex to feet for patients with TCL [19]. 

### 2.1. Prognostic Indicators in T-Cell Lymphoma

The different histological subtypes of PTCL confer different prognoses. Systemic ALCL portends a better prognosis than non-ALCL PTCL [20–23], and within the ALCL subpopulation, those that are ALK-1 positive have a better prognosis than those that are ALK-1 negative (5-year OS, 70% versus 49%) [5]. The international prognostic index (IPI) initially described by Shipp et al. [24] was able to separate patients with PTCL-NOS and systemic ALCL into distinct prognostic risk groups; however, even with low IPI scores, patient with PTCL had poor outcomes [5]. 

A separate prognostic model for patients with PTCL-NOS called the prognostic index for PTCL-U (unspecified); PIT, was proposed [25]. In this retrospective analysis of 385 patients with PTCL-U, multivariate analysis showed that age, performance status, LDH level, and bone marrow involvement were predictive of survival, and these variables were used to develop a new prognostic model that could separate the patients into 4 distinct prognostic groups.

Curiously, neither a high IPI nor PIT score predict for a poorer outcome in AITL [26]. It is postulated that the clinical effects of AITL is due to the dysregulation of the immune system rather than the direct effects of tumour growth, given that hypergammaglobulinemia and eosinophilia accompany this condition, and since IPI and PIT scores generally reflect tumour burden, it was unable to prognosticate in AITL. Furthermore, recent work in gene-expression profiling reveals that AITL is closely associated with follicular helper cells, and that more than 90% of genes in the AITL gene signature were contributed by nonneoplastic cells. The AITL gene signature included genes involved in humoral immune response, recruitment of inflammatory cells, and modulation of vasculogenesis and the extracellular matrix [17]. 

The CTCL have the best 5-year OS; primary cutaneous ALCL: 90% and subcutaneous panniculitis-like PTCL: 64%. EATL and hepatosplenic PTCL are associated with particularly dismal prognoses, with reported 5-year survival rates of only 20% and 7%, respectively.

### 2.2. Gene-Expression Profiling to Better Classify and Risk Stratify PTCL

Even with the revised WHO classification of tumors and the development of several prognostic models, PTCL-NOS remains heterogeneous in terms of biology and outcome. Using microarrays to analyse the gene expression profiles of 59 nodal TCL, Ballester and colleagues have managed to distinguish between AITL, ALCL, and T-lymphoblastic lymphoma, well-characterised entities in the WHO classification [27]. With the same technique, they managed to identify 3 molecular subgroups amongst the PTCL-NOS: PTCL-U1, PTCL-U2, and PTCL-U3. The genes overexpressed in PTCL-U1 included genes associated with a poorer outcome in other tumors such as CCND2 and interestingly, although not significant, the survival of this group was worse than the other 2 groups. PTCL-U2 was associated with genes involved in T-cell activation and apoptosis; NFKB1 and BCL2, and PTCL-U3, with genes involved in the IFN/JAK/STAT pathway, possibly shedding light on the pathogenesis of these tumours. This work, however, is preliminary, and confirmatory studies are needed.

### 2.3. Treatment

No randomized phase III trial has been conducted in PTCL. Thus, by default, Cyclophosphamide, Doxorubicin, Vincristine, and Prednisolone (CHOP)-like chemotherapy, is the most common regimen used despite its lack of efficacy. The international T cell Project confirms the poor prognosis of patients with PTCL treated with CHOP-like regimen [5]. To improve the outcomes of these patients, several strategies have been investigated.

## 3. Increased Dose-Intensity Chemotherapy Regimens

At least two large studies have shown the utility of a dose-intense regimen in improving outcomes in aggressive large-cell lymphomas, including PTCL. The first study compared the ACVBP (doxorubicin, cyclophosphamide, vincristine, bleomycin, and prednisolone) regimen with CHOP in 635 patients aged between 61 to 69 years of age and included 96 (15%) patients with T-cell lymphomas. The dose-intense regimen was shown to significantly improve 5-year event-free survival (EFS) and overall survival (OS) (39% versus 29%, *P* = .005 and 46% versus 38%, *P* = .036, resp.) [28]. The second study was the NHL-B1 trial of the German high-grade non-Hodgkin's lymphoma study group (DHNSL) and it had a 2-by-2 factorial design and compared 2-weekly versus a 3-weekly CHOP with or without Etoposide (CHOEP) in 866 patients under 60 years, of whom 14% had T-cell lymphoma. In pooled analysis, the addition of Etoposide to CHOP chemotherapy in this group of patients significantly improved complete response (CR) rates as well as 5-year EFS (87.6% versus 79.4%, *P* = .003 and 69.2% versus 57.6%, *P* = .004, resp.) although there was no difference in OS [29]. These results were further reinforced in a recent review of all patients with PTCL and NK-cell lymphoma treated prospectively in phase II or III protocols of the DHNSL [30]. Nonetheless, these studies were not designed specifically to address the optimal regimen in patients with PTCL, but suggest that a dose-dense approach is feasible. 

Other attempts at increasing dose density in the setting of PTCL include the use EPOCH (Infusional etoposide, vincristine, and doxorubicin with bolus cyclophosphamide and oral prednisolone) [31] and hyper-CVIDD (hyperfractionated cyclophosphamide, vincristine, dexamethasone, and liposomal doxorubicin) alternated with methotrexate and cytarabine [32] regimens. Peng et al. reported the use of EPOCH in 21 patients with PTCL, the largest study of EPOCH use in PTCL patients. These patients included seven patients with PTCL-U, seven with NKT lymphoma, five with ALCL, one with mycosis fungoides/Sezary syndrome, and another with SPTCL. Of these, seven were previously treated. The overall response rate was 85% (17/20) with 50% (10/20) achieving CR. Grade III/IV neutropenia rate was 34.3% with no treatment-related mortality in this study [31]. In a review of 15 patients with HSTL attending the M.D Anderson Cancer Centre from 1997 to 2007, 4 patients received hyper-CVIDD alternated with methotrexate and cytarabine. Of these, 3 achieved CR and subsequently proceeded with stem-cell transplantation (SCT). Survival was reported as 11, 13, and 14 months for these patients. One patient achieved PR and passed away 8 months later [32].

## 4. High-Dose Chemotherapy and Autologous Stem Cell Rescue

Uncontrolled prospective and retrospective studies suggest that upfront high dose chemotherapy with autologous stem cell rescue (HDC+ASCR) as consolidation in patients who attained complete remission following first-line therapy may result in superior disease control and improved OS rates (Table 3) [33–40]. In a retrospective review of 115 patients with PTCL, the GEL-TAMO group showed that a 5-year OS rate of 80% was achieved in the 37 patients who were transplanted in first CR compared to 45% in those treated in a salvage setting [33]. In another retrospective study of 146 patients with AITL undergoing autologous HSCT, patients transplanted at first remission had significantly better 2-year and 4-year progression-free survival (PFS) compared to those transplanted following chemotherapy-sensitive relapse (2-year PFS, 70% versus 42%; 4 year PFS, 56% versus 30%). The estimated OS rates were also better for those who received a transplant during first CR compared to those transplanted following chemotherapy-sensitive relapse (2-year OS was 81% versus 60%; 4-year OS was 78% versus 47%) [36]. Reimer et al. conducted the largest prospective trial on upfront HDC+ASCR for patients with PTCL [40]. Of the 83 patients enrolled, 55 underwent HDC+ASCR. The estimated 3-year OS rate was 71% for patients who underwent HDC+ASCR compared to only 11% for those who did not. However, this was a single-arm study and about a third of the patients did not undergo HDC+ASCR because of a variety of reasons, including disease progression in 24 (29%). This study highlights two important issues. Firstly, HDC+ASCR following first remission shows promise in improving survival in patients with an estimated 3-year survival rate of 71%. This compares favorably with published data. However, this strategy is limited by the fact that about a third of patients suffered early disease progression. Thus, the challenge is in identifying an effective regimen that can induce a high remission rate so that more patients can undergo HDC+ASCR. (Table 3).

## 5. Graft Versus Lymphoma Effect of Allogeneic Stem Cell Transplantation

Historically, allogeneic SCT is associated with a lower relapse rate than HDC+ASCR in intermediate to high grade lymphomas. The groups that have subsequently attempted to study the role of allogeneic SCT in patients with PTCL have generally found that it is feasible, however, the treatment-associated toxicities are significant. A prospective phase II trial was conducted on 17 patients with relapsed or refractory PTCL, and they were subjected to salvage chemotherapy and reduced-intensity allogeneic SCT [41]. At a median followup of 28 months, 12 patients had CR, 1 had partial response (PR), and 1 had stable disease (SD) status. 3-year OS and PFS were 80% and 64%, respectively. 8 patients (47%) had CMV reactivation, 6 (35%) developed graft versus host disease, and one died of Enterobacter sepsis. In a retrospective review of 77 patients with PTCL who underwent allogeneic SCT, the 5-year OS and event-free survival rates were 57% and 53% respectively [42]. Notably, the 5-year OS for chemoresistant patients was 29%, a result that would not be achieved with conventional approaches alone. Treatment-related mortality, however, was 33%.

## 6. Novel Agents

### 6.1. Anti-Metabolites (Gemcitabine and Pralatrexate)

The addition of anti-metabolites to the treatment of PTCL has been studied. Gemcitabine, a pyrimidine analogue, has been added to a CHOP plus etoposide regimen in a pilot study of 26 patients with newly diagnosed PTCL. The overall response rate was 77% (20 patients), with 16 (62%) achieving complete remission or unconfirmed complete remission (uCR). 14 patients (54%) developed grade IV neutropenia, but there were no treatment-related deaths [43]. 

Pralatrexate is another antimetabolite, which has garnered much interest in the treatment of PTCL. Pralatrexate is an antifolate agent that has high affinity for the reduced folate carrier-1 (RFC-1), whose expression is induced by oncogenes H-ras and c-myc, resulting in selective accumulation of the drug in tumour cells. In a phase I-II study of 51 patients with both pretreated B- and T-cell lymphoma, Pralatrexate was seen to induce a higher overall response rate in the T-cell sub group compared to the B-cell subgroup (54% versus 10%) [44]. The PROPEL (Pralatrexate in patients with relapsed or refractory peripheral T-cell lymphoma) study soon followed as a prospective, phase II, and single-armed study in patients with PTCL who had a median of 3 cycles of chemotherapy. Despite having been heavily pretreated, the overall response rates were a remarkable 27%, with 8% achieving CR or uCR [45]. However, only 12% of patients had a sustained response beyond 14 weeks.

### 6.2. Immunotherapy (Alemtuzumab and Denileukin Diftitox)

Alemtuzumab is a humanized monoclonal antibody against CD52, which is highly expressed on malignant T cells, but also in normal B- and T- lymphocytes [46]. It has been studied in combination with CHOP for the treatment of PTCL in two phase II studies. The first, reported by Gallamini et al., enrolled 24 patients whilst the second by Kim et al., had 20 patients [47, 48]. Both showed high CR rates of 71% and 65%, respectively; however, the both studies documented serious infective complications. In the first study, a quarter of their patients developed life-threatening infections, including invasive aspergillosis, JC viral encephalitis, pneumocystis carinii pneumonia, and suspected tuberculosis [47]. In the second study, febrile neutropenia occurred in 55% of patients while 5 patients, (25%) had CMV reactivation, 3 patients developed CMV disease (pneumonitis or retinitis), and 2 died of treatment-related complications, resulting in the premature closure of the trial [48]. 

Denileukin diftitox (Dd) is a recombinant fusion protein containing the IL-2 (Interleukin-2) protein and a truncated portion of the diphtheria toxin which targets cells expressing the IL-2 receptor. It was approved by the FDA in 2008 for the treatment of CTCL [49] and is now being actively studied for its utility in the treatment of PTCL. In a phase II trial, 27 patients with relapsed/refractory PTCL (CTCL excluded) were treated with single-agent Dd 3 times weekly with tumour staging repeated after every 2 cycles [50]. Six patients (22%) achieved CR and 7 (26%) showed PR. The median PFS was 6 months after a median of 2.5 prior therapies. There were no grade 4/5 toxicities reported. Dd was then combined with CHOP in 49 patients with a 51% CR rate and a median PFS of 12 months [51]. Again, Dd was well tolerated, and multicenter randomized Phase III trials comparing CHOP to Dd-CHOP are in progress.

### 6.3. Histone Deacetylase Inhibitors (HDACis)

Romidepsin was first isolated from the bacteria Chromobacterium violaceum from a soil sample obtained from the Yamagata prefecture in Japan. It had minimal antibacterial activity but showed antitumor effect in human cell lines [52]. 43 patients with relapsed PTCL, treated with a median of 4 previous lines of chemotherapy, were given Romidepsin. The OR rate was 39% (17 patients) and the median duration of response was 8.3 months [53]. Currently, a phase 2b study of Romidepsin in progressive or relapsed PTCL is ongoing at several international centers.

As a class, HDACis are thought to exert their antitumor effect by modulating gene expression thereby influencing cell differentiation or apoptosis. Both Vorinostat, an oral HDACi as well as Romidepsin, have been approved by the FDA for the treatment of CTCL [54, 55].

### 6.4. Conclusion

There is still substantial work to be done in the classification, prognostication, and management of mature T-cell lymphomas. With the advent of high-throughput sequencing, technology is available to quickly identify putative genes involved with each subgroup of TCL, which may allow refinement of the classification and prognostication process. In light of the low incidence of this tumour, international collaboration will be important in accruing patients for larger randomised phase III trials to evaluate the various novel treatment agents.

## Figures and Tables

**Table 1:** List of T-cell lymphomas adapted from the 2008 WHO classification of mature T-cell and NK-cell Neoplasms.

Cutaneous	
(i) Mycosis fungoides	
(ii) Sezary syndrome	
(iii) Primary cutaneous CD30+ T-cell lymphoproliferative disorders	
(iv) Primary cutaneous anaplastic large-cell lymphoma	
(v) Primary cutaneous *γδ* T-cell lymphoma	
(vi) Primary cutaneous CD8+ aggressive epidermotropic lymphoma	
(vii) Primary cutaneous CD4+ small/medium T-cell lymphoma	

Nodal	
(i) Angioimmunoblastic T-cell lymphoma	
(ii) Anaplastic large-cell lymphoma, ALK positive	
(iii) Anaplastic large-cell lymphoma, ALK negative	
(iv) Peripheral T-cell lymphoma, NOS	

Extranodal	
(i) Systemic EBV+ T-cell childhood lymphoproliferative disorder	
(ii) Hydroa vacciniforme-like lymphoma	
(iii) Extranodal NK/T-cell lymphoma, nasal type	
(iv) Enteropathy-associated T-cell lymphoma	
(v) Hepatosplenic T-cell lymphoma	
(vi) Subcutaneous panniculitis-like T-cell lymphoma	

Leukemic	
(i) T-cell prolymphocytic leukaemia	
(ii)T-cell large-granular lymphocytic leukaemia	
(iii) Adult T-cell leukaemia/lymphoma	

**Table 2:** Clinical and histological characteristics of the major PTCL subtypes.

Type of PTCL	Clinical characteristics	Histological features
PTCL-NOS	Most present with peripheral lymph-node enlargement, advanced disease and B symptoms	CD4 > CD8Antigen loss frequent (CD7, CD5, CD4/CD8, CD52)CD 10−, BCL6−, CLCX13−, PD1−

AITL	Patients are middle-aged and elderly and most present with generalised lymphadenopathy, hepatosplenomegaly. Laboratory findings include circulating immune complexes, cold agglutinins, and haemolytic anaemia	CD4+ or Mixed CD4/8 CXCL13+, PD1+Majority BCL6+Half are CD10+ Hyperplasia of follicular dendritic cellsEBV+ Bcell

ALK-positive ALCL	Male predominance (1.5 : 1) and occurs in the first three decades of life. Most patients present with advanced-stage disease, peripheral and/or abdominal lymphadenopathy. They often have B symptoms especially fever	CD 30+ALK translocation is present Majority EMA+ Majority CD2+, CD5+, CD4+, TIA1+, granzyme B+ Majority CD3−, CD8−

ALK-negative ALCL	Compared to ALK+ ALCL, patients are older (40–65 year-old) and the clinical course is more aggressive. Similar to ALK+ ALCL, patients present with advanced disease, peripheral and/or abdominal lymphadenopathy, and B symptoms	CD30+ (strong and homogenous staining) Majority CD3+, CD4+, CD 43+ Minority EMA+PAX5−EBV markers EBER and LMP1 negative

**Table 3:** Selected results of high-dose chemotherapy and autologous stem cell transplantation in PTCL.

Study	*N*	Regimen	Response	OS	Comments
Retrospective series					

Rodríguez et al. [33]	115	BEAM/BEAC/CVB/Cy + TBI	86% CR, 5% PR	56% at 5 y	PTCL
Yamazaki et al. [34]	11	CEM/HD-MTX	91% CR, 9% PR	72% at 3 y	No ALCL
Schetelig et al. [35]	14	Diverse	86% CR	60% at 5 y	AITL only
Kyriakou et al. [36]	146	Diverse	70% CR, 7% PR	59% at 4 y	AITL only

Prospective					

Rodríguez et al. [37]	13	BEAM	65% CR, 4% PR	48% at 3 y	Subgroup analysis
Mercadel et al. [38]	41	High-dose CHOP/ESHAP	51% CR, 7% PR	29% at 4 y	41% received ASCR
D' Amore et al. [39]	77	BEAM	71% CR/PR	No data	75% received ASCR
Reimer et al. [40]	83	Cy/TBI	58% CR, 8% PR	48% at 3 y	66% received ASCR
